# Deterministic fabrication of graphene hexagonal boron nitride moiré superlattices

**DOI:** 10.1073/pnas.2410993121

**Published:** 2024-09-27

**Authors:** Rupini V. Kamat, Aaron L. Sharpe, Mihir Pendharkar, Jenny Hu, Steven J. Tran, Gregory Zaborski, Marisa Hocking, Joe Finney, Kenji Watanabe, Takashi Taniguchi, Marc A. Kastner, Andrew J. Mannix, Tony Heinz, David Goldhaber-Gordon

**Affiliations:** ^a^Department of Physics, Stanford University, Stanford, CA 94305; ^b^Stanford Institute for Materials and Energy Science, SLAC National Accelerator Laboratory, Menlo Park, CA 94025; ^c^Department of Materials Science and Engineering, Stanford University, Stanford, CA 94305; ^d^Research Center for Functional Materials, National Institute for Materials Science, Tsukuba 305-0044, Japan; ^e^Department of Physics, Massachusetts Institute of Technology, Cambridge, MA 02139

**Keywords:** moiré superlattice, graphene, boron nitride

## Abstract

The electronic properties of moiré heterostructures depend sensitively on the relative orientation between layers of the stack. For example, near-magic-angle twisted bilayer graphene (TBG) commonly shows superconductivity, yet a TBG sample with one of the graphene layers rotationally aligned to a hexagonal Boron Nitride (hBN) cladding layer provided experimental observation of orbital ferromagnetism. To create samples with aligned graphene/hBN, researchers often align edges of exfoliated flakes that appear straight in optical micrographs. However, graphene or hBN can cleave along either zig-zag or armchair lattice directions, introducing a $30^{{}^{\circ}}$ ambiguity in the relative orientation of two flakes. By characterizing the crystal lattice orientation of exfoliated flakes prior to stacking using Raman and second-harmonic generation for graphene and hBN, respectively, we unambiguously align monolayer graphene to hBN at a near-0^°^, not 30^°^, relative twist angle. We confirm this alignment by torsional force microscopy of the graphene/hBN moiré on an open-face stack, and then by cryogenic transport measurements, after full encapsulation with a second, nonaligned hBN layer. This work demonstrates a key step toward systematically exploring the effects of the relative twist angle between dissimilar materials within moiré heterostructures.

## Significance

Moiré heterostructures, including the canonical twisted bilayer graphene, host exotic correlated electronic states such as superconductivity and ferromagnetism. These states depend sensitively on heterostructure details, including the relative twist of all component materials. In twisted bilayer graphene, it has been shown that alignment of a graphene layer with the proximate hBN cladding layer can result in orbital magnetism. However, reproducing such phenomena has proven challenging due to the difficulty in precisely setting the relative twist between two dissimilar, yet isostructural van der Waals materials. Here, we lay out a general process for reliably stacking aligned graphene-hBN heterostructures and rapidly verifying structural parameters mid-fabrication through scanned probe microscopy, en route to electronic transport measurements after full encapsulation and fabrication.

With their reliably calculable band structure and high degree of tunability, moiré heterostructures have demonstrated a great variety of strongly correlated and topological phases. Experimentally realizing these exotic phases requires precisely controlling the relative orientation of constituent layers of the heterostructure. In moirés where each layer is the same material, such as the canonical twisted bilayer graphene (TBG), angular control to within ∼0.1^°^ of a targeted angle is enabled by “tear and stack” or “cut and stack” techniques, in which one crystalline exfoliated flake is bisected and the two sections are then stacked with the desired interlayer twist angle (1, 2). This technique is important since TBG with interlayer twist within $0.1^{{}^{\circ}}$ of the $1.1^{{}^{\circ}}$ “magic angle” typically features correlated states at integer fillings of the moiré flat bands (3), and superconductivity at nearby fillings (4–7).

Moiré patterns can also form between dissimilar but isostructural materials. For such heterobilayers, alignment can have a strong effect on the structure’s electronic properties, yet precisely setting the interlayer twist angle is challenging. In cut-and-stacked homobilayers, the two layers are known to begin with the same orientation before an intentional twist is introduced, but in heterobilayers such a starting zero-twist reference is not available without additional characterization.

Consider the materials pair of graphene and hexagonal boron nitride (hBN), whose lattice spacings differ by only ∼1.8%. Alignment to hBN can dramatically change the electronic properties of graphene-based stacks, whether monolayer graphene (8–14), Bernal bilayer graphene (15, 16), twisted bilayer graphene (17, 18), or multilayer rhombohedral graphene (19–22). In this work, we are particularly motivated by the impact of hBN alignment on ground states of TBG near the magic angle. Of the many near-magic-angle TBG devices that have been fabricated and thoroughly characterized worldwide over the past five years, only two, to our knowledge, have instead demonstrated a robust orbital ferromagnetic state at 3 electrons per moiré unit cell at zero magnetic field (17, 18). In each of these devices, one of the hBN cladding layers was aligned to within 1^°^ with the proximate graphene layer. Of the two TBG devices, one exhibited quantized Hall resistance at zero magnetic field and $n/n_{s}=3$ (18), indicating that this ferromagnetic state is a quantum anomalous Hall (QAH) state.

What causes QAH in TBG, and why has it been so rarely observed? The nearly flat conduction and valence minibands of TBG are connected by Dirac points at the corners of the mini Brillouin zone, which are protected by $C_{2z}T$, where $C_{2z}$ is xy-inversion symmetry and T is time reversal symmetry. Achieving QAH requires breaking both. Breaking $C_{2z}$ gaps the Dirac points, and breaking T then allows uneven filling of the fourfold spin- and valley-degenerate copies of the moiré flat band. Each copy has nonzero Chern number ±1, so filling an odd number of copies gives a net Chern number and thus a quantum-Hall-like state.

Initial theories for QAH in near-magic-angle TBG implied that the hBN-graphene alignment observed in the two ferromagnetic TBG devices was necessary and sufficient to break $C_{2z}T$ (23, 24): hBN lacks $C_{2z}$ symmetry so aligning monolayer graphene to hBN breaks $C_{2z}$ (9, 25) in the graphene, while flat band electron–electron interactions spontaneously break T. However, these theories did not account for the graphene–hBN lattice mismatch, which causes the inversion symmetry in graphene to be broken only locally at periodically spaced AA stacking sites in the graphene–hBN moiré. Subsequent theory of QAH in near-magic angle TBG suggests a more stringent condition on the structure: commensurability between the coexisting TBG and graphene–hBN moirés (26–28). The simplest commensurability occurs when the two moirés share the same period and orientation, so that the entire three-layer system forms a single moiré pattern. This criterion is satisfied only at specific pairs of twist angles, notably a graphene–hBN twist angle of ±0.6^°^ and a graphene–graphene twist angle of ^±^1.2^°^ (26, 29).

Recent STM studies suggest that local graphene–graphene–hBN commensurability occurs over a broader range of twist angles than predicted for rigid lattices, indicating that it may be energetically favorable (29). Still, in practice, we are unlikely to realize perfectly commensurate moiré structures globally in such a 3-layer system. Fortunately, theory suggests that QAH should be observable by transport even in devices with one or both twist angles up to ∼0.1^°^ off from the ideal (in which case a “supermoiré” or “moiré of moirés” is formed) (26).

In short, precise, reliable, and verifiable control over the relative angle between graphene and hBN is necessary (and likely sufficient) for reproducing and further investigating QAH in TBG. It should also allow the exploration of novel states in other twist-controlled moiré heterostructures built from graphene and hBN, or from a wider range of layered materials including transition metal dichalcogenides. To date, rotationally aligning graphene with other isostructural van der Waals materials has relied on visually aligning long (O(10μm) or longer) “straight” edges of exfoliated materials. These edges often (but not always) result from cleavage along high-symmetry planes (30, 31) of the crystal lattice, and thus can serve as a proxy for the crystallographic orientation of the flakes (13). The crystallinity of an edge is often corroborated by the presence of multiple straight edges differing in orientation by integer multiples of $30^{{}^{\circ}}$. However, such cleavage can occur along two distinct crystallographic directions, yielding straight edges with zigzag or armchair termination. Though simulations and experiments on suspended graphene membranes have suggested that graphene shows a preference for ripping along armchair edges (32, 33), in practice both types of edges are frequently observed in exfoliated flakes of graphene and hBN. Thus, visually aligning straight edges in hBN and graphene can accidentally match a zigzag graphene edge with an armchair hBN edge or vice versa, resulting in 30^°^ misalignment. Furthermore, even seemingly straight edges can be noncrystallographic and contain some mixture of zigzag and armchair structure (34, 35).

Here, we outline a general approach for fabrication and rapid verification of Van der Waals heterostructures with defined relative twist angle. We demonstrate this method on graphene/hBN structures, but it should work quite broadly for other pairs of exfoliatable materials. Our process flow is a generalization of what is already used for heterobilayers made from two different transition metal dichalcogenides (TMDs), where second-harmonic generation optical spectroscopy is already well-established as a guide for setting the relative orientation of the two layers (36–39). We use second harmonic generation spectroscopy on hBN flakes and Raman spectroscopy on graphene flakes to 1) confirm whether straight edges of graphene and hBN flakes are crystallographic, and 2) identify crystallographic edges as either zig-zag or armchair, eliminating ambiguity in how exfoliated graphene–hBN flakes should be oriented during stacking to form a moiré superlattice. We then use torsional force microscopy (TFM) (40) not only to verify near-alignment of graphene and hBN, but also to serve as a transport-independent real-space probe of moiré structural parameters. Finally, we use low-temperature transport measurements to verify the existence of the graphene–hBN superlattice and extract a moiré unit cell area consistent with the TFM measurements performed prior to encapsulation of the graphene. In sum, these techniques enable stacking dissimilar materials at near-aligned twist angle and rapidly confirming the existence of an associated moiré at intermediate fabrication steps.

## 1. Results

We first use second harmonic generation spectroscopy to characterize the orientation of hBN exfoliated flakes. In any noncentrosymmetric crystal, illumination with a laser at frequency f causes emission at 2f, a phenomenon known as second-harmonic generation (SHG). The strength of the second harmonic signal depends on the laser polarization relative to the lattice directions of the crystal, so measuring the polarization dependence allows determining the crystal orientation. Samples that are noncentrosymmetric and thus susceptible to this method for determining orientation include atomic monolayers or flakes of odd number of layers of TMDs or hBN (41, 42), as well as Bernal-stacked trilayer graphene (43). This method is commonly used when preparing to stack two different TMD monolayers to form a heterobilayer with a specific twist angle (36–39). More salient for our purposes, a small but measurable SHG signal has also been reported in *many-layer* hBN flakes, regardless of layer number parity: Nonnegligible thickness of the hBN flakes compared to the laser wavelength (1060 nm in our case) causes a gradient in the electric field strength, breaking c-axis inversion symmetry in the light–sample interaction and thus allowing quadrupole contributions to the SHG signal (15, 44).

For hBN, the orientation dependence is described by a sixfold symmetric pattern $I_{\left|\right|}=I_{0}\cos^{2}\left(3\theta \right)$, where θ is the angle between the laser polarization $\vec{P}$ and a mirror plane of the crystal, and $I_{\left|\right|}$ is the component of the second harmonic signal polarized parallel to the pump laser polarization (41).

**Fig. 1.**
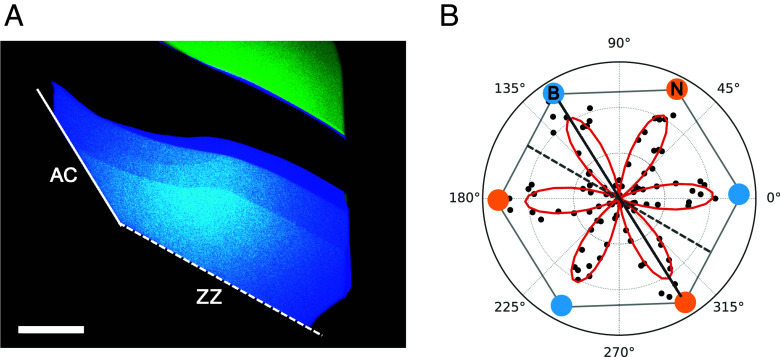
Polarization-resolved SHG of many-layer hBN. (*A*) Optical image of an hBN flake. Solid and dashed white lines indicate straight edges of interest. (Scale bar: 20 μm.) (*B*) SHG signal intensity as a function of laser polarization. The straight edge indicated by the dashed (solid) white line in the optical image lies along a node (maximum) in the polarization-resolved SHG data marked by a dashed (solid) gray line, indicating a zigzag (armchair) edge. Blue and yellow dots schematically represent the orientation of the boron and nitrogen, respectively, of the hBN crystal lattice expected from the measured SHG response. Exchanging orientations of B and N would be just as consistent with the six-fold-symmetric SHG pattern. As-grown hBN crystals are AA’ stacked, so the orientation of B and N swaps with each successive atomic layer.

Consistent with prior reports (15), we observed SHG and the expected sixfold dependence on polarization in all 15+ many-layer exfoliated hBN flakes of 30 nm to 60 nm thickness we studied—see Fig. 1 for one example—though roughly half were presumably even-layered and thus centrosymmetric; see Fig. 1 for one such example. The orientation of minima and maxima in the SHG intensity as a function of polarization is used to identify straight edges of the flake as either armchair or zigzag. Armchair edges in hBN are parallel to a mirror plane of the lattice, whereas zigzag edges are oriented between mirror planes of the lattice. Thus, a crystalline edge for which $\vec{P}$ parallel to the edge yields a node (maximum) in the SHG signal is a zigzag (armchair) edge. Edges which do not line up with either a node or a maximum in the SHG signal are noncrystallographic, and hence not useful for establishing orientation.

Additional SHG measurements are shown in *SI Appendix*, Fig. S11. As noted above, SHG signals have also been observed in Bernal ABA-stacked trilayer graphene, which is noncentrosymmetric (43). Thus, SHG could be used to determine crystal orientation for some graphene-based structures, in preparation for stacking with defined twist angle. However, *monolayer* graphene, which we intend to align with hBN in a stack, is centrosymmetric and so does not produce a SHG signal. Its orientation must be characterized in another way.

**Fig. 2.**
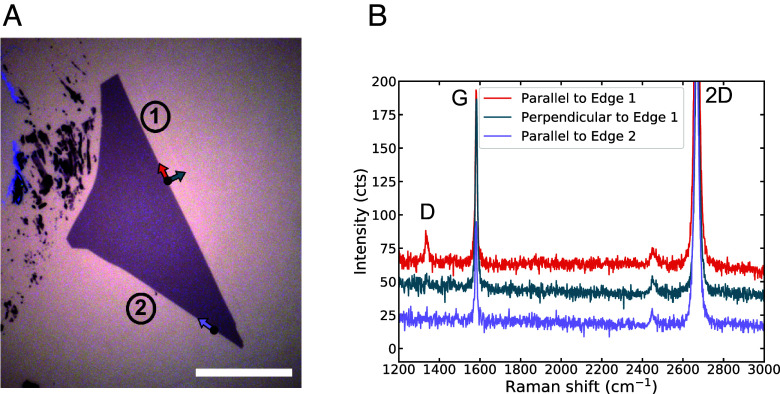
Polarized Raman on monolayer graphene. (*A*) Optical image of an exfoliated graphene flake with straight edges, separated by a 30^°^ corner. (Scale bar: 20 μm.) (*B*) Raman spectra taken at the edges of the flake. Location of laser spot and direction of laser polarization for each spectrum are indicated by the solid black points and colored arrows in (*A*). The D peak on Edge 1 that vanishes when the laser is polarized perpendicular to the edge indicates that edge 1 of (*A*) is likely armchair. Edge 2 is offset from edge 1 by 30^°^, and shows no D peak signal, so is likely zigzag.

To determine the crystallographic nature of straight edges in exfoliated graphene flakes, we use polarized Raman spectroscopy. The D peak at ∼1,350 cm^−1^ in Raman spectroscopy of graphene (Fig. 2*B*) is absent in the interior of a pristine graphene flake like those produced by exfoliation from a high-quality graphite crystal. This peak originates from a two-step intervalley scattering process (34): inelastic scattering of the excited electron (or hole) with a phonon, then elastic scattering off of a feature that breaks translation symmetry, such as a point defect or sample edge (*SI Appendix*, Fig. S8*A*). Because a uniform edge only transfers momentum along its normal vector, a perfect zigzag edge cannot scatter electrons between different valleys, whereas an armchair edge efficiently induces intervalley scattering (*SI Appendix*, Fig. S8*B*). Thus, in an exfoliated monolayer graphene flake we expect to observe a D peak only near an armchair edge (or an edge containing armchair segments). Even for an armchair edge, the intensity of the D peak strongly depends on the excitation laser polarization (30, 34, 35, 45): maximal for laser polarization parallel to the edge, minimal—zero for an ideal edge—for polarization perpendicular to the edge. The ratio of D peak intensities for the two orientations can provide a measure of edge disorder, such as microscopic segments of differing termination (35).

On a single crystal graphene flake, if two edges differ in orientation by an odd integer multiple of 30^°^ (Fig. 2*A*), and each edge has well-defined termination, then one edge must be zigzag and the other armchair. Indeed, in Raman spectra taken at each edge with the laser polarization oriented parallel to the given edge, we observe a clear D peak at the *Right* edge and no D peak on the *Bottom Left* edge (Fig. 2*B*). Rotating the laser polarization to be perpendicular to the *Right* edge, we see that the D peak disappears. This indicates that the *Right* edge (Edge 1) has armchair edge termination, and the *Left* edge (Edge 2) has zigzag edge termination.

Similar characterization performed on other graphene flakes can be seen in *SI Appendix*, Figs. S9 and S10. In ideal cases, as exemplified by flakes in Fig. 2 and *SI Appendix*, Figs. S5 and S9, particular edges can be unambiguously assigned as zigzag or armchair. However in some flakes (three out of the eight flakes measured for this work), edges separated by an odd integer multiple of 30^°^ both exhibit a D peak (*SI Appendix*, Fig. S10). This can occur when an edge either is not crystallographic or is oriented macroscopically along a zigzag axis but contains microscopic armchair segments, preventing unambiguous assignment of edge termination. To target specific relative alignment of graphene and hBN in a stack, we use only graphene flakes whose edge terminations we can definitively assign.

Having directly identified the crystallographic termination of straight edges on a graphene flake and an hBN flake, we can fabricate aligned graphene–hBN heterostructures by visually aligning straight edges of now-known termination during stacking, offsetting by 30^°^ if we are pairing a zigzag edge with an armchair one. Using standard dry transfer techniques (*Methods*), we first pick up the hBN flake shown in Fig. 1*A*. We then rotationally align the zigzag edge of the hBN flake (now on the stamp) with the zigzag edge of the graphene flake in Fig. 2*A*, and pick up the graphene flake. With the aligned graphene–hBN heterostructure now on the polymer stamp, we use TFM to characterize the moiré.

TFM is a local probe technique which uses a torsional resonance mode of an AFM cantilever to probe changes in local dynamic friction, reliably imaging open-face graphene–graphene and graphene–hBN moirés (40). The technique is nondestructive and can be applied to a stack still mounted on a polymer stamp. After TFM imaging, encapsulation can be completed, the stack dropped onto a substrate, and standard lithography used to pattern a device for transport measurements.

**Fig. 3.**
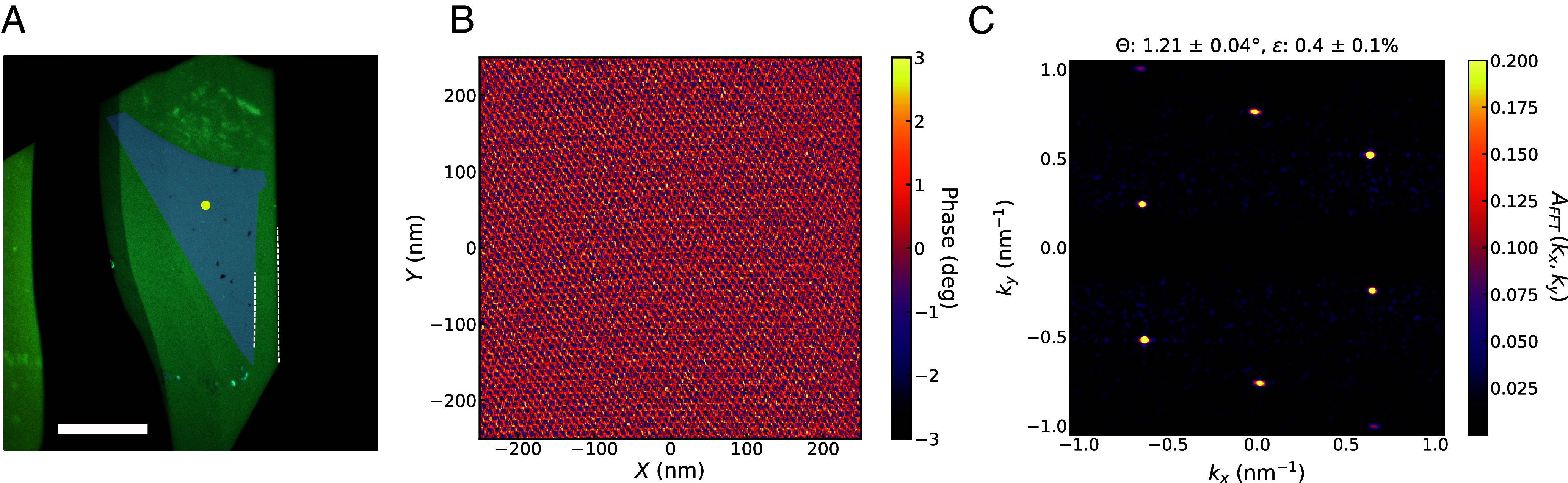
Torsional Force Microscopy of graphene–hBN moiré. (*A*) Optical image of the aligned graphene–hBN stack on a polymer stamp. In preparation for stacking, the zigzag edge of the graphene flake from Fig. 2*A* (false colored in purple) was visually aligned with the zigzag edge of the hBN flake from Fig. 1. Dashed white lines indicate the zigzag edges of each flake. The yellow dot indicates rough location of TFM scan. (Scale bar: 20 μm). (*B*) Phase component of the TFM signal over a 500 nm × 500 nm scan. (*C*) FFT of TFM scan in (*B*). Moiré parameters are extracted from FFT peak positions by fitting to a simple model with twist angle (θ) and uniaxial heterostrain (ϵ) as fit parameters.

A 500 nm × 500 nm TFM scan on our aligned graphene–hBN stack shows a clear moiré superlattice in the phase component of the TFM signal (Fig. 3). A Fourier transform of these data yields a set of sharp peaks associated with the periodicities of the moiré superlattice. Using a simple model with twist angle and uniaxial heterostrain as fit parameters, we extract from the peak positions an estimated graphene–hBN misalignment of $1.21\pm 0.04^{{}^{\circ}}$ and uniaxial heterostrain of 0.4±0.1% (Fig. 3*B*). We do not correct for thermal drift and piezo actuator creep in the TFM measurement, which can distort the image of the moiré superlattice and lead to an overestimation of strain but should not as strongly influence the extracted twist angle. These effects will be examined more closely in upcoming work.

Using the same process flow described here, we fabricated a second graphene–hBN heterostructure, shown in *SI Appendix*. For that stack, TFM images yield a graphene–hBN twist angle of ∼1.9^°^, again (as intended) close to $0^{{}^{\circ}}$ rather than $30^{{}^{\circ}}$, demonstrating that the spectroscopic characterization correctly identifies the crystallographic axes of both graphene and hBN.

Following TFM characterization, the graphene–hBN stack is deposited onto an annealed hBN-graphite gate heterostructure (previously assembled and deposited onto a SiO_2_/Si substrate), and patterned into two Hall bars (*Methods*). The bottom hBN flake is deliberately misaligned with respect to the aligned graphene–hBN, to ensure that no moiré is formed with the bottom hBN.

**Fig. 4.**
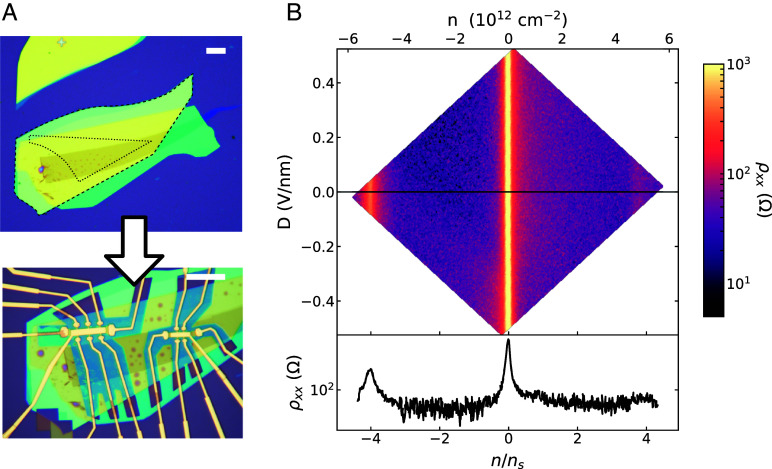
Transport characterization of graphene–hBN moiré at 1.5 K. (*A*) *Top* optical image shows the hBN-encapsulated monolayer graphene heterostructure prior to patterning. Dashed black outlines indicate the borders of the precharacterized graphene and hBN flakes; the hBN flake being the larger of the two. The stack is patterned into two Hall bars (*Bottom* optical image), one with a doped Si back gate (*Left*) and the other with a graphite back gate (*Right*). Each Hall bar has a metal *Top* gate. (Scale bars: 20 μm.) (*B*) *Top*—Color map of longitudinal resistivity in the *Left*-hand Hall bar of (*A*), as a function of carrier density n and displacement field D. *Bottom*—Line cut of resistance vs. carrier density along D=0. The resistance peak at n=−5.15e12 cm^−2^ indicates full emptying of the graphene–hBN moiré superlattice ($n/n_{s}=-4$).

Graphene aligned to hBN exhibits peaks in resistivity not only at charge neutrality but also at fillings of 4 holes and/or 4 electrons per moiré unit cell. Here we observe such a peak at density n=−5.15E12±0.05E12 cm^−2^. Associating this with 4 holes per moiré unit cell yields a unit cell area 77.7 ± 0.8 nm^2^, corresponding to a graphene–hBN twist angle of $1.10^{{}^{\circ}}\pm 0.01^{{}^{\circ}}$. The error bars here are dominated by uncertainty in gate capacitance, which is calibrated by fitting the slopes of features in the Landau fan diagram (Fig. 4*C*). The locations of gaps in density and magnetic field follow the Diophantine relation: For integer s and t, $n/n_{s}=t\left(\phi /\phi_{0}\right)+s$, where $n_{s}$ is the carrier density corresponding to 4 electrons per moiré unit cell, ϕ is the magnetic flux per moiré unit cell, and $\phi_{0}=h/e$ is the magnetic flux quantum. In this sample, gaps we observe correspond to (s=0,−4) and (t=±2,6,10,14,...).

**Fig. 5.**
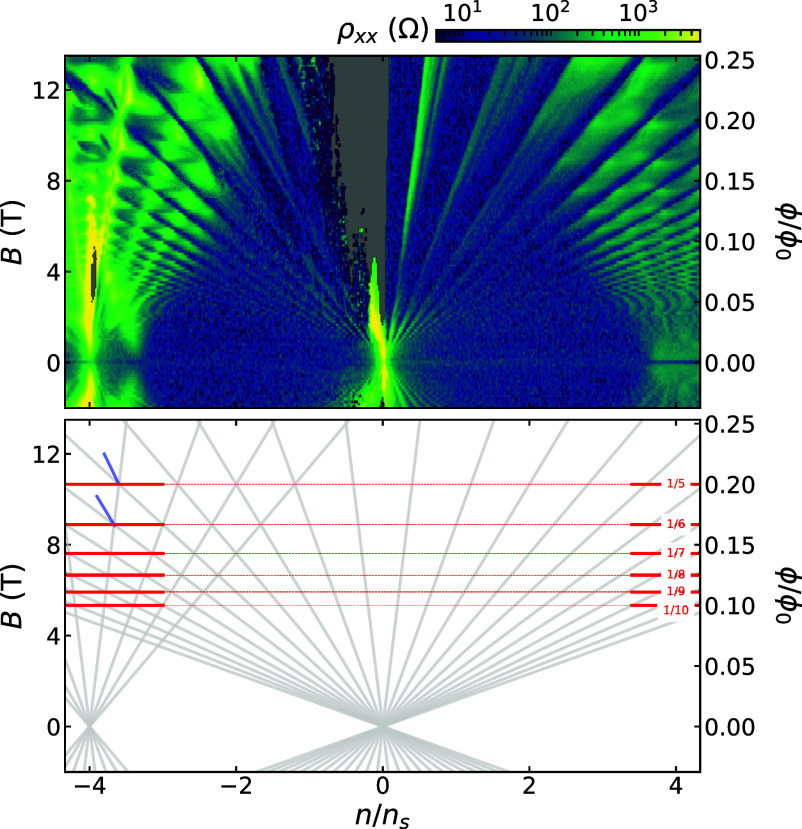
Wannier diagram of graphene–hBN moiré at 1.5 K. *Top*—Landau fan of longitudinal resistivity at D=0, for the same Hall bar as in Fig. 4*B*. *Bottom*–Hofstadter energy spectrum features observed in the Landau fan measurement. Gaps in the fractal Hofstadter energy spectrum emanating from charge neutrality (s=0, t=±2,6,10,14,...) and from the graphene–hBN superlattice miniband edge (s=−4, t=±2,6,10) are indicated by gray lines. At magnetic fields where these gaps intersect ($\phi /\phi_{0}=1/m$ for m=5,6,7,8,9) are local minima in $\rho_{\mathrm{xx}}$, marked by red lines (clearest at higher $n/n_{s}$). Indications of broken-symmetry states with (s,t)=(−2,−8) and (−2,−10) are marked by blue lines.

We also measure clear Brown-Zak oscillations (46) (Fig. 5). Though these carrier-density-independent features can in principle occur at any rational value of flux per moiré cell, we measure them at $\phi /\phi_{0}=1/m$ for integer m, where gaps from different s intersect. The magnetic field values at which these features occur are a direct measure of moiré unit cell area, independent of gate capacitance. In this sample, we thereby extract a unit cell area of 77.7 ± 0.4 nm^2^, corresponding to a graphene–hBN twist angle of $1.10^{{}^{\circ}}\pm 0.06$. Here, error bars are set by the width of the oscillations in magnetic field.

In addition to the Brown-Zak oscillations and Landau fan features emanating from charge neutrality and $n/n_{s}=\pm 4$, we see a high resistance feature emanating from $n/n_{s}=-3.3$ both in this device and in the secondary device shown in *SI Appendix*, Fig. S2. At this same filling of the moiré band, the Hall density diverges (*SI Appendix*, Fig. S1), likely marking a Van Hove singularity (VHS) predicted near that filling (47). A similar high longitudinal magnetoresistance feature, near a noninteger moiré band filling, has been reported in a doubly aligned hBN/graphene/hBN moiré (48). Such magnetoresistance may be caused by electron correlations combined with high density of states near a VHS, or by open Fermi surfaces that occur in the presence of a small amount of uniaxial heterostrain, which breaks the degeneracy of a VHS (49).

Measurements of the second Hall bar produced from this same heterostructure are shown in *SI Appendix*. The average moiré cell area in the second Hall bar is 58.0 nm^2^, corresponding to a graphene–hBN twist angle of 1.4^°^. This indicates a substantial variation (0.3^°^) in the graphene–hBN twist between different locations in the heterostructure. Spatial variation in twist angle is generically observed in moiré heterostructures. Variation this large may appear somewhat surprising in an annealed graphene/hBN moiré, a type of sample which is typically assumed to be particularly robust against twist angle disorder. However, this stack contained many bubbles (*SI Appendix*, Fig. S4). Though each Hall bar was defined in a relatively bubble-free region, the two Hall bars were separated by bubbles, giving an opportunity for twist angle to vary between the Hall bars. The TFM measurements only covered a 500 nm × 500nm area, and so did not capture the full range of moiré structure present across an entire Hall bar, let alone both Hall bars. Still, the TFM-extracted twist angle (∼1.2^°^) is consistent with the transport-extracted twist angle range of 1.1^°^ to 1.4^°^. This indicates that the moiré superlattice likely did not change dramatically during encapsulation, device fabrication, or cryogenic cool-down. This validates in-process TFM as a tool to determine superlattice period, select regions of relatively uniform period for device fabrication, and inform analysis of transport measurements on completed encapsulated devices.

## 2. Discussion

We have combined multiple techniques to enable reliable formation and validation of low-twist-angle graphene–hBN moirés. First, we used optical spectroscopy techniques to identify the crystallographic orientation of both graphene and hBN, guiding the rotation angle chosen to match the orientation of the two flakes as they are stacked. After stacking these two flakes but before encapsulation with a second hBN layer, we used TFM to verify the existence of a moiré and characterize its period. Edge assignment determined through SHG on hBN was also independently confirmed through atomic-resolution TFM (*SI Appendix*, Fig. S3). After encapsulation and nanofabrication of Hall bars, we performed cryogenic transport measurements and found that the moiré unit cell area extracted from transport measurements agrees reasonably well with that measured by TFM prior to encapsulation and nanofabrication.

The same edge precharacterization and stacking process was followed for a second open-faced graphene–hBN aligned stack described in detail in *SI Appendix*. TFM measurements on this stack showed a graphene–hBN twist angle of 1.9^°^. Transport measurements were not performed on this stack. The fact that both heterostructures fabricated showed graphene–hBN alignment close to 0^°^, not 30^°^, indicates that the likelihood of accidental alignment is relatively low.

There are some limitations to this process. First, the techniques we use are effective for binary assignment of straight edges as either zigzag or armchair, but are not usable on flakes lacking straight edges or flakes whose apparently straight edges are disordered/noncrystallographic. The latter seems common in graphene, where we see a number of flakes with apparently straight edges that produce an inconclusive D-peak signal (*SI Appendix*, Fig. S10). This does not limit our reliability in correctly aligning graphene to hBN when we stack the two together—we simply do not proceed with stacking of graphene flakes featuring such apparently noncrystallographic edges. Still, in our experience with exfoliation, straight edges are less common on monolayer graphene flakes than on tens-of-nm-thick hBN flakes, and the prevalence of noncrystallographic/disordered straight edges on graphene further lowers the proportion of graphene flakes which are suitable for alignment with hBN. In the future, ambiguity in graphene edge termination might be addressed with atomic-lattice-resolution AFM-based measurements such as TFM (40) or conductive AFM on an appropriate substrate (50). Graphene flakes of different thicknesses are often found attached to each other or closely spaced following exfoliation, in which case the orientation has been found to be preserved (or nearly so) between flakes (48). Should monolayer graphene happen to be found attached to or near a thicker flake, optical spectroscopy [e.g., SHG on Bernal trilayer graphene (43)] or atomic-resolution TFM of the thicker region could thus be used to infer orientation of the monolayer.

Another limitation is a lack of precise control over the final graphene–hBN twist angle. In the device presented above, we targeted a 0^°^ twist angle during stacking, but TFM and transport measurements indicate a twist angle ranging from $1.1^{{}^{\circ}}$ to $1.4^{{}^{\circ}}$ in different regions of the heterostructure. These measured twist angles agree with the angle between zigzag edges in AFM images of the stack (*SI Appendix*, Fig. S4), to the accuracy with which we can extract the angle from those AFM images. This indicates that the observed misalignment is not from inaccurate characterization of lattice orientation, but instead from imprecise setting of initial alignment and/or shifting of flakes during dry transfer pickup. This is reminiscent of our experience fabricating TBG using tear- or cut-and-stack methods, where, despite guaranteed initial alignment between the two layers, the final observed twist angle in transport often differs from the intended angle by tenths of degrees or even more.

The degree of alignment we achieved so far is not sufficient for systematically exploring electronic phases of hBN-aligned TBG. A proposed criterion for observing a QAH state in such a system is close proximity to a specific pair of commensurate angles (26–28), demanding setting both the graphene–graphene angle and the graphene–hBN angle to within 0.1^°^. Reaching this benchmark will require significant improvement in our initial alignment and/or stacking techniques, which will be the subject of further work.

Though work remains to improve the accuracy of the target angle, as explained above, we have taken major steps toward more reliable and repeatable fabrication of graphene–hBN moirè superlattices. We have also integrated TFM measurements as an accurate and noninvasive technique for characterizing the graphene–hBN twist angle mid-device fabrication. This in-process characterization provides two important advantages: 1) Filtering out stacks which do not have the intended twist angle before time is invested in lithography and transport measurements, and 2) Serving as a transport-independent probe of device structure, including local information on not just twist angle but also heterostrain, enabling more systematic study of the connection between structural parameters and low-energy electronic phases.

## 3. Methods

Graphene and hBN flakes are prepared by standard mechanical exfoliation with Scotch magic tape onto a 300 nm SiO_2_/Si substrate, annealed at 90 C to 110 C for several minutes.

Polarized Raman measurements are taken on a Horiba XploRA+ Confocal Raman system using a 532 nm excitation laser and a 2,400 gr/mm grating. The laser is focused through a 100× objective lens (0.9 numerical aperture) for a nominal laser spot size of 376 nm. Raman maps shown in *SI Appendix* are performed with a step size of 0.4 μm. Default laser polarization of the tool is oriented in the sample plane, along the vertical axis. Alignment of graphene flake edges with laser polarization is achieved by rotating the sample with the use of a precise, 360^°^ manual rotation stage. The laser polarization can be rotated 90^°^ by a half-wave plate, enabling efficient acquisition of polarized Raman spectra both parallel and perpendicular to a given graphene edge.

SHG measurements are performed on a home-built SHG setup. The excitation laser is a femtosecond pulsed laser (NKT Origami Onefive 10) with wavelength 1,030 nm and pulse duration <200 fs. Collection is done with an Andor iXon Ultra EMCCD, which measures the component of the material’s SHG response polarized parallel to the excitation laser.The excitation laser polarization is rotated from 0^°^ to 180^°^ by a Union Optics Super Achromatic half wave plate. The half wave plate has its own nonnegligible SHG signal, visible in raw data shown in *SI Appendix*, which is removed via background subtraction when fitting the SHG response of hBN flakes.

The aligned graphene-on-hBN stack in the main text is prepared using the standard dry transfer technique for assembling vdW structures. A glass slide with a thin Poly(Bisphenol A carbonate) film over a gel (Gel-Pak DGL-17-X8) stamp is brought into contact with the previously exfoliated and SHG-characterized hBN flake, which is heated to ∼80 ^°^C. After successfully picking up of the hBN flake, the hBN flake is lowered into contact with a precharacterized exfoliated graphene flake, with careful alignment of the zigzag edges of both flakes as determined by SHG and polarized Raman measurements.

Moiré morphology characterization was performed on the open-faced graphene–hBN-stamp assembly in a Bruker Dimension Icon with a Nanoscope 5 controller. Measurements are performed with an Adama Innovations AD-2.8-SS conductive diamond tip on a PF-TUNA cantilever holder in torsional excitation mode. Procedures are described in extensive detail in ref. 40.

hBN above a graphite backgate is separately assembled with dry-transfer techniques, deposited onto a 300 nm SiO_2_/doped Si substrate, and annealed for 3 h at 500 ^°^C in an Ar/O_2_ atmosphere to remove polymer residue. The aligned graphene–hBN stack is then deposited on this graphite backgate stack, encapsulating the graphene. In this step, the graphene is deliberately misaligned with the bottom hBN. The final heterostructure is then washed in solvent and again annealed for 500 ^°^C in an Ar/O_2_ atmosphere before being patterned into two Hall bars, one using the graphite backgate and one using the doped Si substrate as a backgate, for transport measurements. A lithographically defined Ti/Au topgate layer is deposited for both Hall bars, and the mesa is etched with a CHF_3_/O_2_ etch (50 sccm/5 sccm) before depositing Cr/Au one-dimensional edge contacts.

Transport measurements are performed at a base temperature of 1.5 K in a CRYO Industries Variable Temperature Insert with a 14T Oxford Instruments magnet.

When fitting the Wannier diagram (Fig. 5 and *SI Appendix*, Fig. S2), there are two free parameters: the moiré unit cell area and the conversion of voltage to superlattice filling. The unit cell area is obtained from fitting the Brown-Zak oscillations to $\phi /\phi_{0}=1/n$ for integer n. We then identify the gate voltage corresponding to full-filling ($n/n_{s}=\pm 4$) as the gate voltage to which the corresponding Landau levels extrapolate at zero field. We ignore quantum capacitance corrections. Once these two parameters are fit, any features in the experimental data that line up well with Steda lines in the Wannier diagram can be assigned their matching (s, t) values. For example, the dark blue line segments in the *Bottom* panel of Fig. 5 are assigned (s, t) = (−2, −8) and (−2, −10) in this way.

## Supplementary Material

Appendix 01 (PDF)

## Data Availability

The data from this study are available at the Stanford Digital Repository (51).
