# Supplementary material for: Deterministic fabrication of graphene hexagonal boron nitride moiré superlattices
Source: Proc Natl Acad Sci U S A. 2024 Sep 27;121(40):e2410993121. doi: 10.1073/pnas.2410993121 (PMC11459135; doi:10.1073/pnas.2410993121)
Supplement: Supplementary file 1 — Appendix 01 (PDF) [file pnas.2410993121.sapp.pdf]

PNAS
www.pnas.org

Supplementary Information for

Deterministic fabrication of graphene hexagonal boron nitride moiré superlattices

Rupini V. Kamat, Aaron L. Sharpe, Mihir Pendharkar, Jenny Hu, Steven J. Tran, Gregory Zaborski Jr., Marisa Hocking, Joe Finney, Kenji Watanabe, Takashi Taniguchi, Marc. A. Kastner, Andrew J. Mannix, Tony Heinz, and David Goldhaber-Gordon

Aaron Sharpe
E-mail: aaron.sharpe@stanford.edu
Marc Kastner
Email: mkastner@mit.edu
David Goldhaber-Gordon.
E-mail: goldhaber-gordon@stanford.edu

This PDF file includes:

Supplementary text
Figs. S1 to S11 (not allowed for Brief Reports)
SI References

Supporting Information Text

1. Additional Transport Measurements

The second of the two Hall bars shown in Figure 4A was also measured in the VTI at 1.5K. The transport measurements of this second device also show signatures of a graphene-hBN moiré superlattice (Figure S2).

These signatures include a weak but present resistance feature at finite hole density, corresponding to $n/n_s = -4$, diagonal Landau fan features emanating from $n/n_s = -4$, and Brown-Zak oscillations with a $\propto 1/B$ periodicity.

However, the transport features of this second device differ in detail from those shown in Figure 5. First, the resistance peak corresponding to four holes per graphene-hBN moire unit cell is weaker and broader and occurs at a higher density than the one observed in the main text (roughly 6.9E12 cm^{-2} carrier density versus 5.15e12 cm^{-2} carrier density). Second, the Brown-Zak oscillations corresponding to $\phi/\phi_0 = 1/m$ (m being some integer) also occur at magnetic field values different from those in the main text. Both of these indicate a difference in average moire unit cell area between the two devices, with an estimated twist angle difference of roughly 0.3° (1.1° average twist angle in device 1 and 1.4° average twist angle in device 2).

This level of twist variation over a length scale of tens of microns is not surprising, but this difference in transport behavior from two devices fabricated on a shared graphene-hBN heterostructure underlies ongoing challenges in using Van der Waal materials for systematic and reproducible study of novel electron transport. The long-distance variation of the graphene-hBN superlattice across the heterostructure was not explored; TFM measurements covered only a 500 nm x 500 nm area (Figure 3). Though more time consuming, large area TFM scans can be used in the future to both inform device placement and provide more detailed structural information to clarify transport behavior as a function of position in micron scale devices.

2. Atomic-Resolution Torsional Force Microscopy of hBN

Atomic-resolution TFM was also performed on the hBN flake displayed in main text Figure 1, after assembly of the fully encapsulated heterostructure as well as fabrication of the two Hall bar devices. The atomic scale TFM reveals a triangular lattice of "bright spots" which we interpret as the B-N atomic bonds. The FFT of the TFM image thus reveals the orientation of the atomic lattice. The orientation of the atomic lattice in the TFM scan independently confirms the edge assignment determined through polarization-resolved SHG measurements on the same hBN flake.

3. Atomic Force Microscopy of Stack 1

After the full heterostructure in the main text was assembled, AFM measurements were taken in a Park XE 70 instrument to determine hBN thickness and to visually inspect post-stacking alignment of the pre-characterized graphene and hBN flakes (Figure S4). The final stack had several bubbles, but the zigzag edges of the pre-characterized graphene and hBN flakes could still be identified and their relative orientation roughly measured as $1.2^\circ \pm 0.1^\circ$.

The angle between edges that were identified as crystallographic zigzag edges in optical pre-stack characterization agrees with the twist angle measured in TFM, and falls within the range of twist angles extracted from transport measurements on the two Hall bars. This further validates pre-stack characterization techniques used.

4. Aligned graphene-hBN Stack 2 Data

An additional graphene-hBN stack was fabricated using the same optical pre-characterization and TFM post-characterization techniques in the main text. This stack was not encapsulated and fabricated into a device for transport measurements, but still provides further validation for the process flow outlined in the main text.

The graphene flakes used in this stack is shown in Figure S5, along with the polarized Raman pre-characterization results. The flake labelled flake 2 was characterized with 2D Raman map, showing where on the flake the D peak signal was observed most prominently. Flakes 1 and 2 share a common vertical edge, and are confirmed to have the same crystal orientation.

The hBN flake used in this stack is shown in Figure S6, along with the SHG characterization results.

The graphene flakes 1 and 2 are used to create two separate graphene-hBN heterostructures on the same hBN flake using standard dry transfer techniques. First, the hBN flake brought into contact with flake 1, with an armchair edge of flake 1 carefully aligned with an armchair edge of the hBN flake. The hBN flake is then lifted away, picking up graphene flake 1 via van der Waals attraction, forming an aligned graphene-hBN heterostructure and leaving the remaining graphene flakes on the substrate. The substrate is then rotated 30° and a separate region of the hBN flake is brought into contact with flake 2, now with a zigzag edge of the hBN flake aligned with an armchair edge of flake 2, for deliberate 30° misalignement. The Poly(Bisphenol A carbonate) film with the graphene/hBN stack on it is then carefully removed from the PDMS gel/glass slide stamp and placed on top of a SiO_2/Si chip to create a open-faced graphene/hBN sample. The chip is then heated to 180 °C for 2 minutes, during which the polymer film flattens and adheres to the SiO_2/Si substrate.

The two graphene-hBN heterostructures are then characterized with TFM (Figure S7). The deliberately misaligned graphene-hBN heterostructure showed no moiré structure, as expected, while the aligned graphene-hBN heterostructure showed a clear moire, validating our armchair/zigzag assignment of graphene and hBN edges.

We take an FFT of the TFM image and identify the position of six peaks corresponding to the moiré reciprocal lattice vectors. These can be fit to a simple model (described below) to determine twist angle and uniaxial heterostrain in the graphene-hBN stack. Like the stack described in the main text, this is a larger twist angle than we'd expect, given that the intended twist angle was zero degrees. However in this case the twist angle error can be in part attributed the fact that the crystal facets that

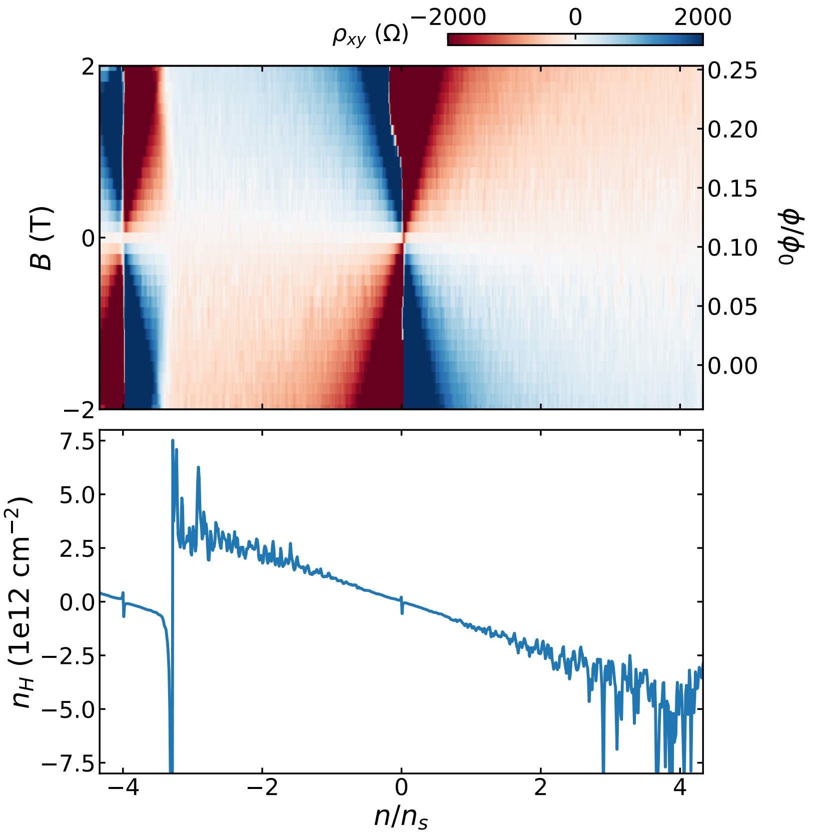

Fig. S1. Hall resistance and density in Device 1 Top - Hall resistance as a function of field and carrier density at D = 0. Bottom - Calculated hall density (n_H) as a function of carrier density. In addition to Lifshitz transitions at charge neutrality and at the graphene-hBN superlattice band edge at $n/n_s = 4$, we see a divergence in the Hall density accompanying a change in the sign of the hall resistance at $n/n_s = -3.3$. This lines up with a high resistance feature in the longitudinal magnetoresistance of this device. This likely indicates a Van Hove Singularity (VHS) in the superlattice band structure at $n/n_s = -3.3$.

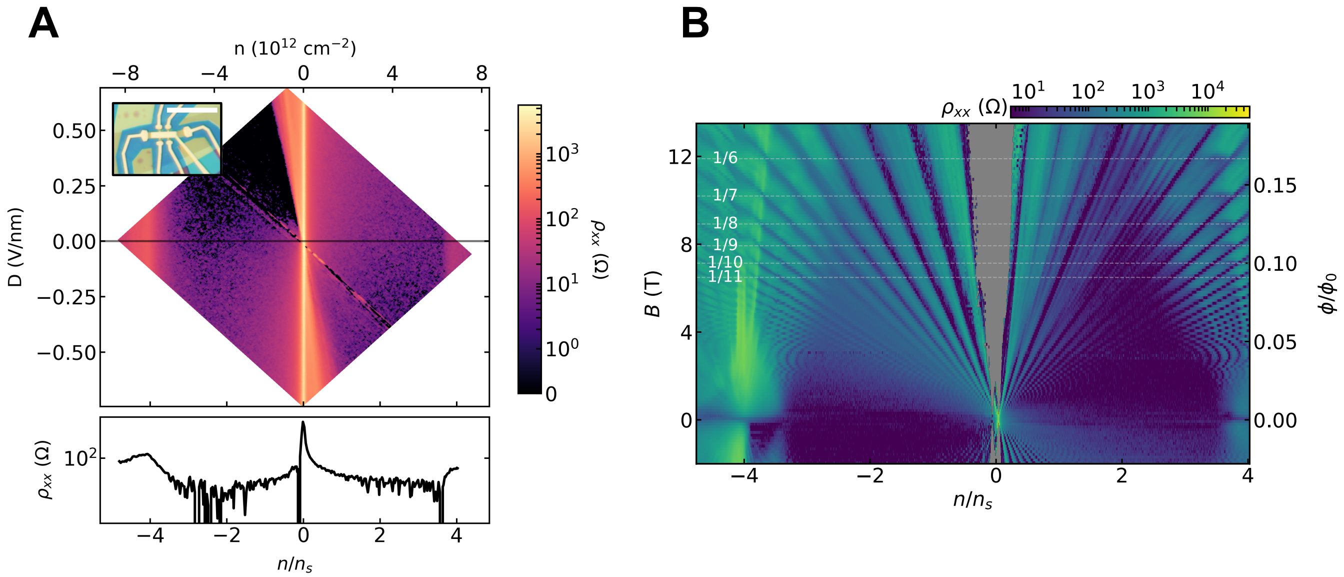

Fig. S2. Device 2 Transport Results A) Longitudinal resistivity (ρ_{xx}) as a function of carrier density n and displacement field D. A broad peak at $n \sim 6.9e12$ cm^2 corresponds to four holes per moiré unit cell ($n/n_s = -4$). **B)** Longitudinal resistivity as a function of normalized carrier density n/n_s and magnetic field B. In addition to Hofstadter energy spectrum gaps corresponding to ($s = 0$, $t = \pm 2, 6, 10, 14...$) and (faintly) ($s = -4$, $t = \pm 2$), there are horizontal features corresponding to $\phi/\phi_0 = 1/m$ for $m = 6, 7, 8, 9, 10, 11$, which provide a measure of the moiré unit cell area.

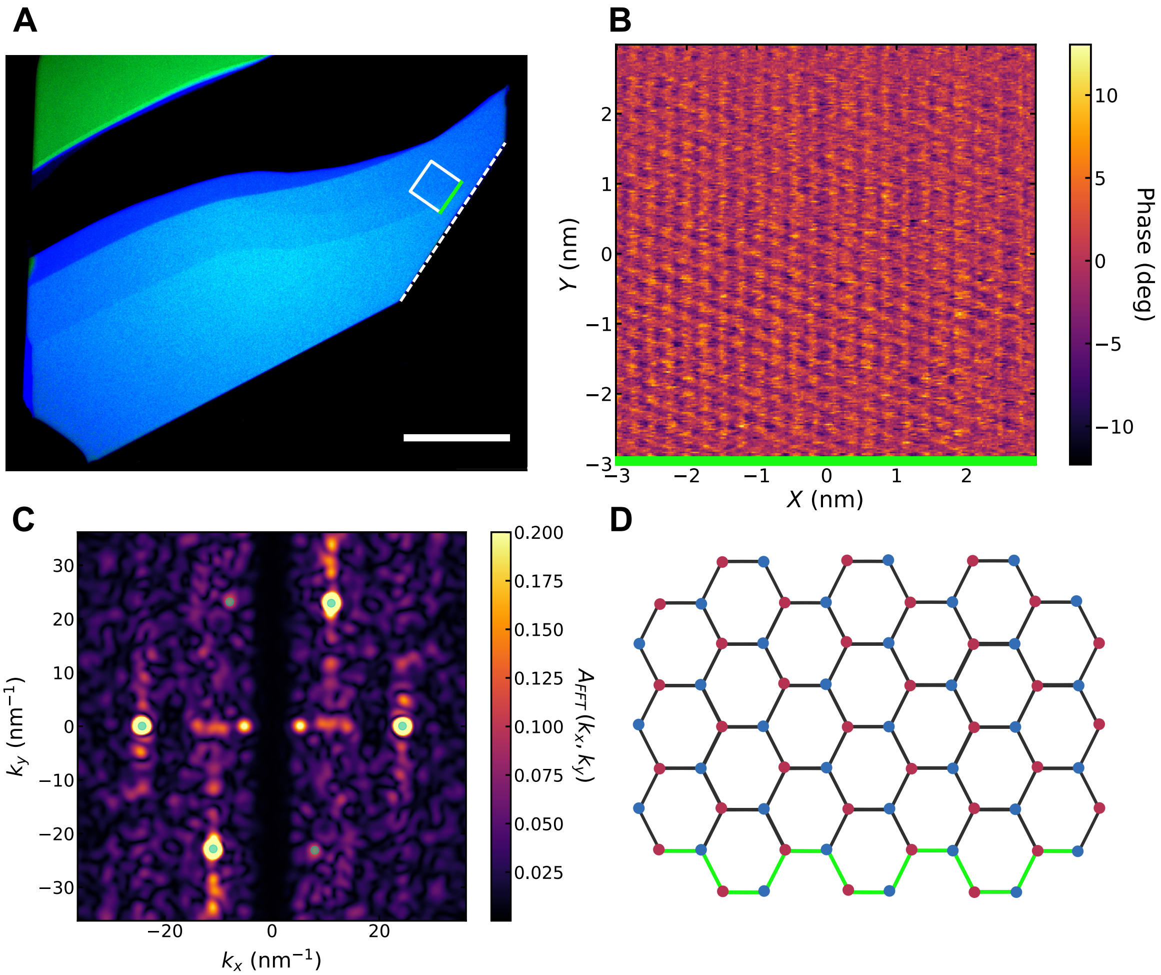

Fig. S3. Atomic-resolution TFM of hBN flake A) Optical image of hBN flake prior to stacking and device fabrication. White square shows the rough location and orientation of the TFM scan, with the size being greatly exaggerated and the green line indicating the bottom of the TFM scan. Scale bar is 20 μm. **B)** 6 nm $\times$ 6 nm TFM scan shows the hBN atomic lattice. **C)** FFT of the TFM scan in **B**). Main peaks are identified with light blue spots. **D)** Expected atomic lattice orientation using FFT peaks identified in **C**, which confirms that the edge indicated by the solid white line in **A** has an armchair termination.

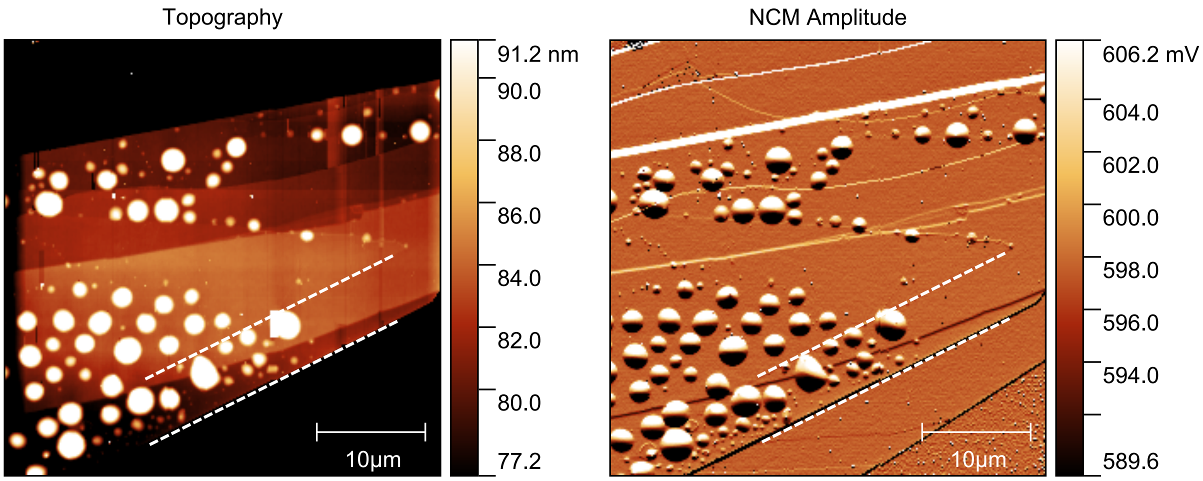

Fig. S4. AFM of Stack 1 Topography (left) and Amplitude (right) from a tapping mode AFM measurement of the stack described in the main text. The graphene and hBN edges which were identified as zigzag edges with polarized Raman and SHG measurements respectively, and visually aligned during stacking, are indicated by the white dashed lines. These lines are measured to have a $\sim 1.2^{\circ}$ angle between them.

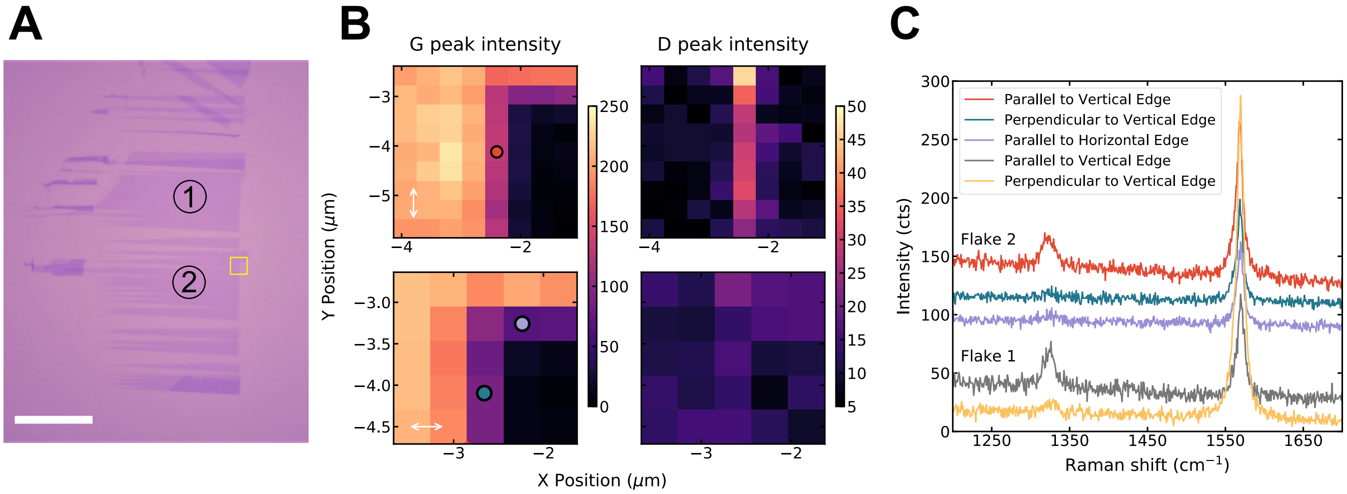

Fig. S5. Polarized Raman Characterization for Stack 2 A) Optical micrograph of two exfoliated flakes, labelled flake 1 and flake 2. Scale bar is 20 μm. **B)** Raman maps showing G peak (left) and D peak (right) intensities in the region indicated with the yellow outline in A, where flake 2 has a small 90° corner. Two different maps are taken, one with vertically polarized light (top) and one with horizontally polarized light (bottom) indicated by double headed white arrows. **C)** Raman spectra taken on both flake 1 and flake 2. The spectra shown from flake 2 are pulled from the map in B - the location of each spectra are indicated by correspondingly colored dots. Two spectra taken along flake 1's vertical edge are also shown (for vertically and horizontally polarized light) to confirm that the two flakes share a crystallographic edge.

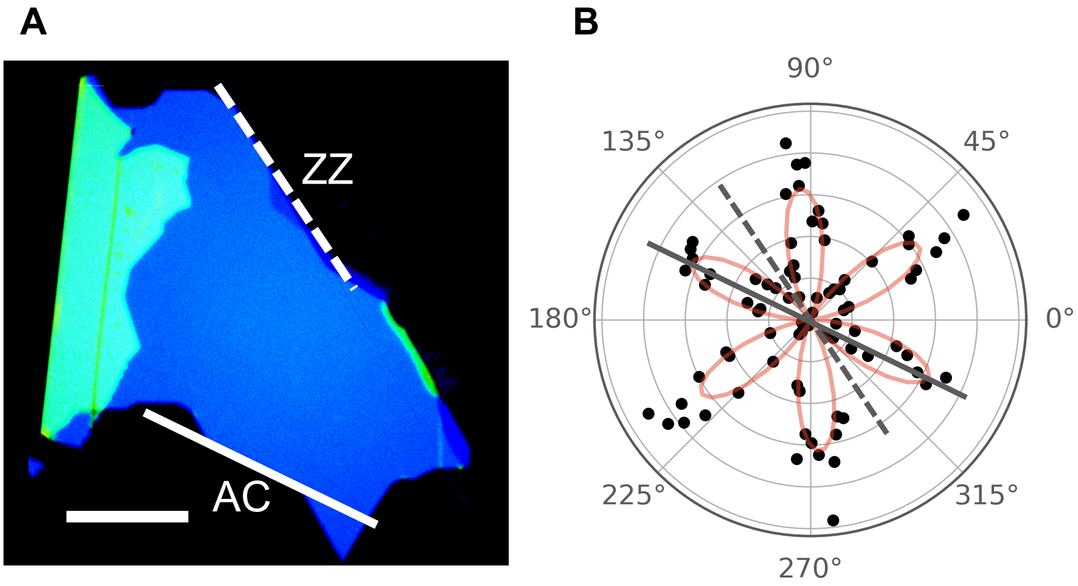

Fig. S6. SHG Characterization for Stack 2 A) Optical micrograph of hBN flake, with straight edges of interest indicated by white lines. Scale bar is 20 μm. **B)** Polarization-resolved SHG results taken on this flake. The polarization orientation corresponding to a maximum (node) in the SHG signal intensity are indicated by a solid (dashed) grey line, and are used to label edges in A as armchair (zigzag).

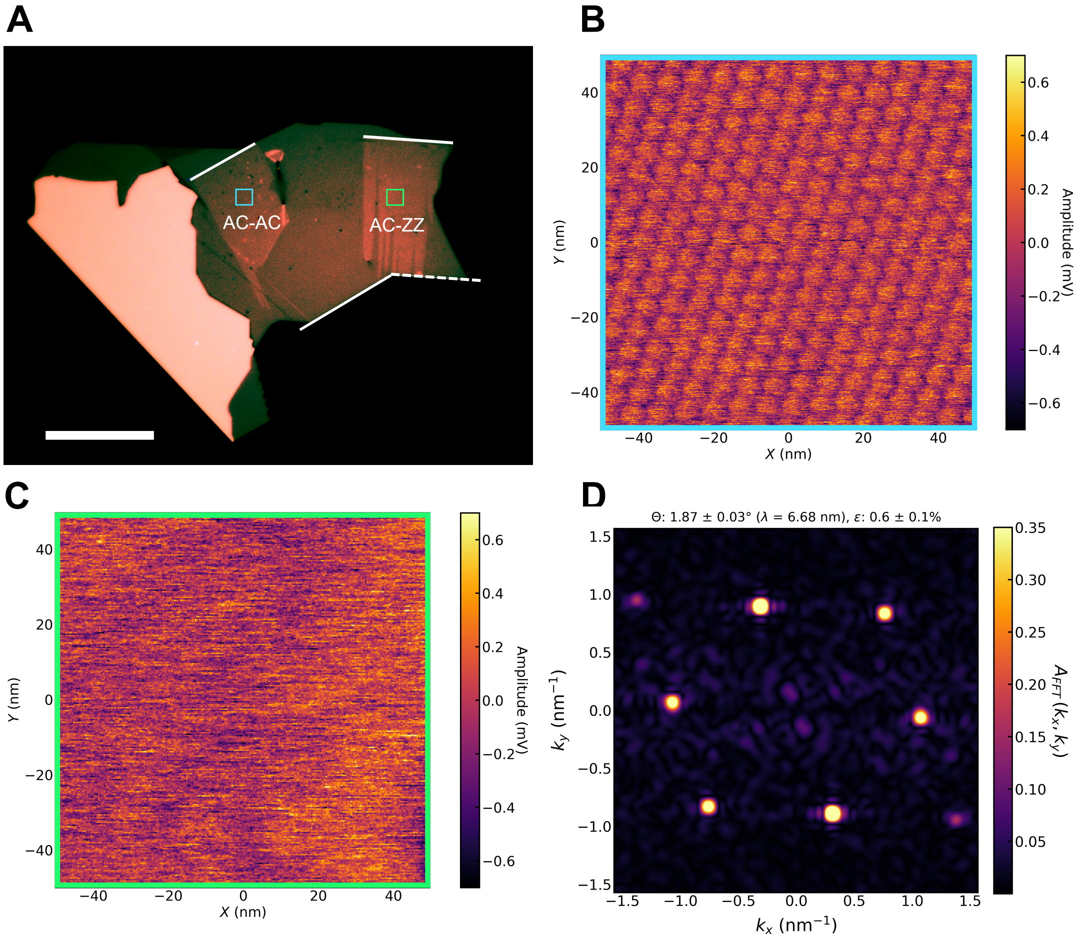

Fig. S7. TFM Post-Stacking Characterization for Stack 2 A) Optical micrograph of stack 2 on a polymer stamp. Two graphene flakes are visible on the hBN - the armchair edge of the left (right) flake was aligned with an armchair (zigzag) edge of the hBN to form a deliberately aligned (misaligned) heterostructure. Armchair (zigzag) edges used for alignment are indicated by solid (dashed) white lines. **B)** TFM scan of aligned graphene-hBN heterostructure. Rough location of the 100 nm x 100 nm scan indicated by blue box in A. A clear moiré is present. **C)** TFM scan of misaligned graphene-hBN heterostructure. Rough location of the 100 nm x 100 nm scan indicated by green box in A. No moiré is visible on this scale. **D)** FFT of TFM image in B. Locations of peaks are use to fit a simple model using twist angle θ and uniaxial heterostrain ϵ as fit parameters. Fit results are indicated by blue dots.

were aligned are relatively short (under 10 microns), so angular misalignment that would be more obvious with longer straight edges were missed when the facets were aligned by eye during stacking. This source of error can be mitigated in the future by using a high magnification, high resolution optical image to carefully measure graphene and hBN edge orientations with respect to a common long, flat surface before stacking, to reduce human error in alignment during stacking.

5. FFT Peak Fitting

To extract moiré parameters from FFT peak positions, we calculate the expected FFT peak positions (corresponding to the two moiré reciprocal lattice vectors and their sum) as a function of some initial guess of the following parameters: global crystal orientation (Ψ), relative twist (θ), and uniaxial heterostrain magnitude (ϵ) and direction (ϕ). We then use a least squares fitting method, which steps through the parameter space until the calculated FFT peak positions match the peaks shown in Figure 3C and Supplementary Figure S7C, to determine our heterostructure parameters.

To calculate expected FFT peak positions, we start with the unit cell lattice vectors for graphene and hBN: $\vec{a_1} = (a, 0)$ and $\vec{a_2} = (-a/2, \sqrt{3}a/2)$, where a is the the lattice constant 2.46 Å for graphene and 2.50 Å for hBN. The hBN lattice vectors are then rotated by the global angle Ψ, and the graphene lattice vectors are rotated by both Ψ and θ as well as 'strained' by some magnitude ϵ along some direction ϕ. These transformations are performed via matrix multiplication with the matrices below.

Rotation matrix for both rotation by arbitrary angle α:

$$R(\alpha) = \begin{pmatrix} \cos(\alpha) & -\sin(\alpha) \\ \sin(\alpha) & \cos(\alpha) \end{pmatrix} \quad \text{[S1]}$$

Uniaxial heterostrain matrix:

$$S(\epsilon, \psi) = I + R(\psi)^T \begin{pmatrix} \epsilon & 0 \\ 0 & -0.16\epsilon \end{pmatrix} R(\psi) \quad \text{[S2]}$$

$S(\epsilon, \psi)$ stretches the lattice by a factor ϵ along direction ψ, and contracts it by 0.16ϵ perpendicular to ψ, where 0.16 is the estimated Poisson ratio of graphene. Note - experimental measurements of the Poisson ratio of graphene have measured values ranging from 0.13 to 0.19(1–4).

After transforming the graphene and hBN lattice vectors, the reciprocal lattice vectors of the individual lattices are calculated using the simple expressions

$$\vec{b}_1 = 2\pi \frac{R(\frac{\pi}{2})\vec{a}_2}{\vec{a}_1 \cdot R(\frac{\pi}{2})\vec{a}_2} \quad \text{[S3]}$$

$$\vec{b}_2 = 2\pi \frac{R(\frac{\pi}{2})\vec{a}_1}{\vec{a}_2 \cdot R(\frac{\pi}{2})\vec{a}_2} \quad \text{[S4]}$$

The moiré reciprocal lattice vectors are then calculated using the difference between the graphene and hBN reciprocal lattice vectors:

$$\vec{b}_{M1} = \vec{b}_{g1} - \vec{b}_{hBN1} \quad \text{[S5]}$$

$$\vec{b}_{M2} = \vec{b}_{g2} - \vec{b}_{hBN2} \quad \text{[S6]}$$

The six FFT peak positions are thus given by $\pm\vec{b}_{M1}$, $\pm\vec{b}_{M2}$, and $\pm(\vec{b}_{M1} - \vec{b}_{M2})$.

After making some initial guess of fit parameters Φ, θ, ϵ, and ψ, a least squares fit searches the parameter space until the calculated moiré reciprocal lattice vectors match the peak positions in the FFT of the TFM scan. The reported uncertainty values come from the Jacobian matrix reported by the least squares fit algorithm from the scipy.optimize library.

6. Polarized Raman Additional Information

Polarized Raman characterization results for a number of additional graphene flakes are shown here. The results shown in Figure S9 enable unambiguous assignment of edges to armchair and zigzag edge terminations, while the results shown in Figure S10 are less clear.

7. Second Harmonic Generation Additional Data

Polarization resolved second harmonic generation measurements were performed on several hBN flakes, the results of which are shown in Figure S11.

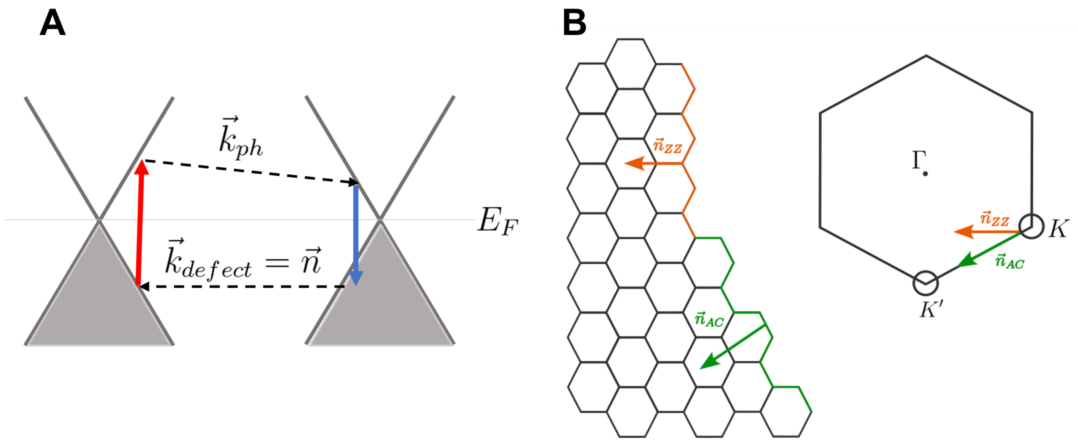

Fig. S8. Edge-induced Double Resonant Raman Scattering in Graphene. A) Schematic of the double resonant Raman scattering process behind the D peak in graphene's Raman spectrum. Red (blue) arrow indicates carrier excitation (relaxtion) via photon absorption (emission). Dashed lines indicate scattering of the excited carrier with a phonon ($\vec{k}_{ph}$) or a defect/edge ($\vec{k}_{defect} = \vec{n}$). B) Crystal structure of graphene and momentum transfer direction associated with scattering off a zigzag (orange) or armchair (green) edge. Edge normal vectors are super-imposed on the graphene Brillioun zone, showing that armchair edges can facilitate intervalley scattering while zigzag edges cannot.

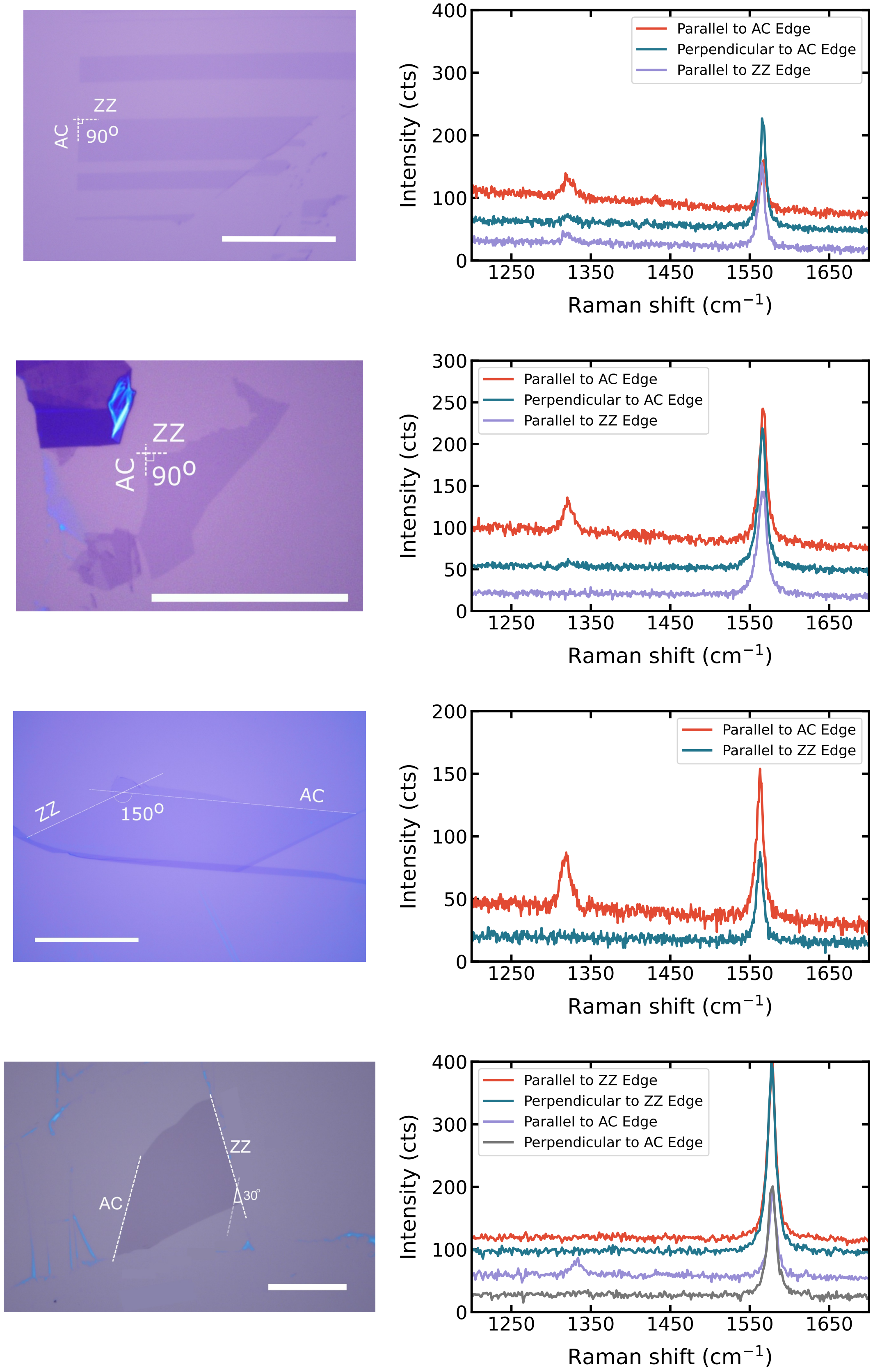

Fig. S9. Additional Examples of Successful Polarized Raman Characterization Four different monolayer graphene flakes, each with at least two straight edges separated by an odd integer multiple of 30°. For each of these flakes, one of the identified straight edges shows a prominent D peak signal for laser polarization oriented along the edge, while the other does not. This allows for clear identification of armchair and zigzag edges. In three out of the four flakes, the D peak signal disappears when the laser polarization is rotated perpendicular to the edge, indicating a low disorder edge.

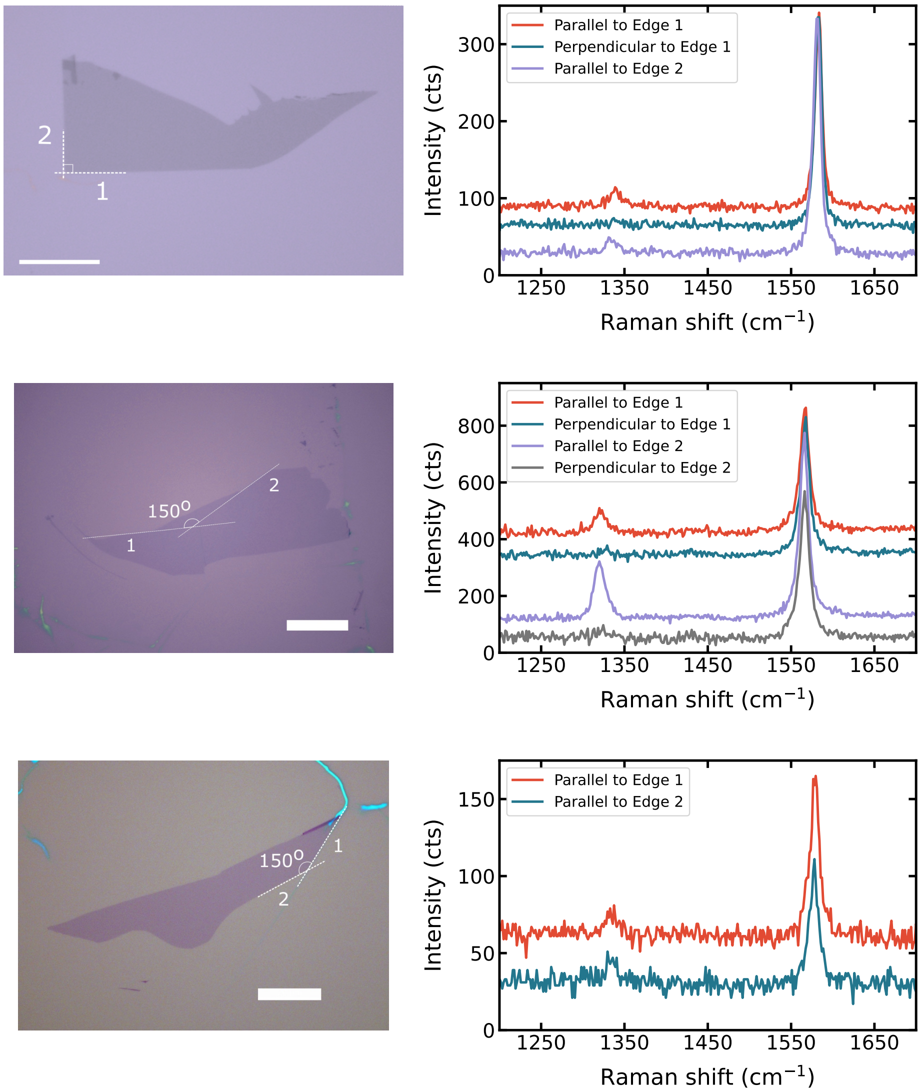

Fig. S10. Examples of Ambiguous Polarized Raman Characterization Three different monolayer graphene flakes, also with at least two straight edges separated by an odd integer multiple of 30°. Though the edges would appear to be crystallographic, Raman characterization of the flakes yielded ambiguous results, with both of the indicated edges showing some indication of a D peak signal. This is likely be the result of zigzag edges that are highly disordered and contain armchair segments that yield a D peak, but it is also possible that that the apparently straight edges indicated here are not crystallographic at all.

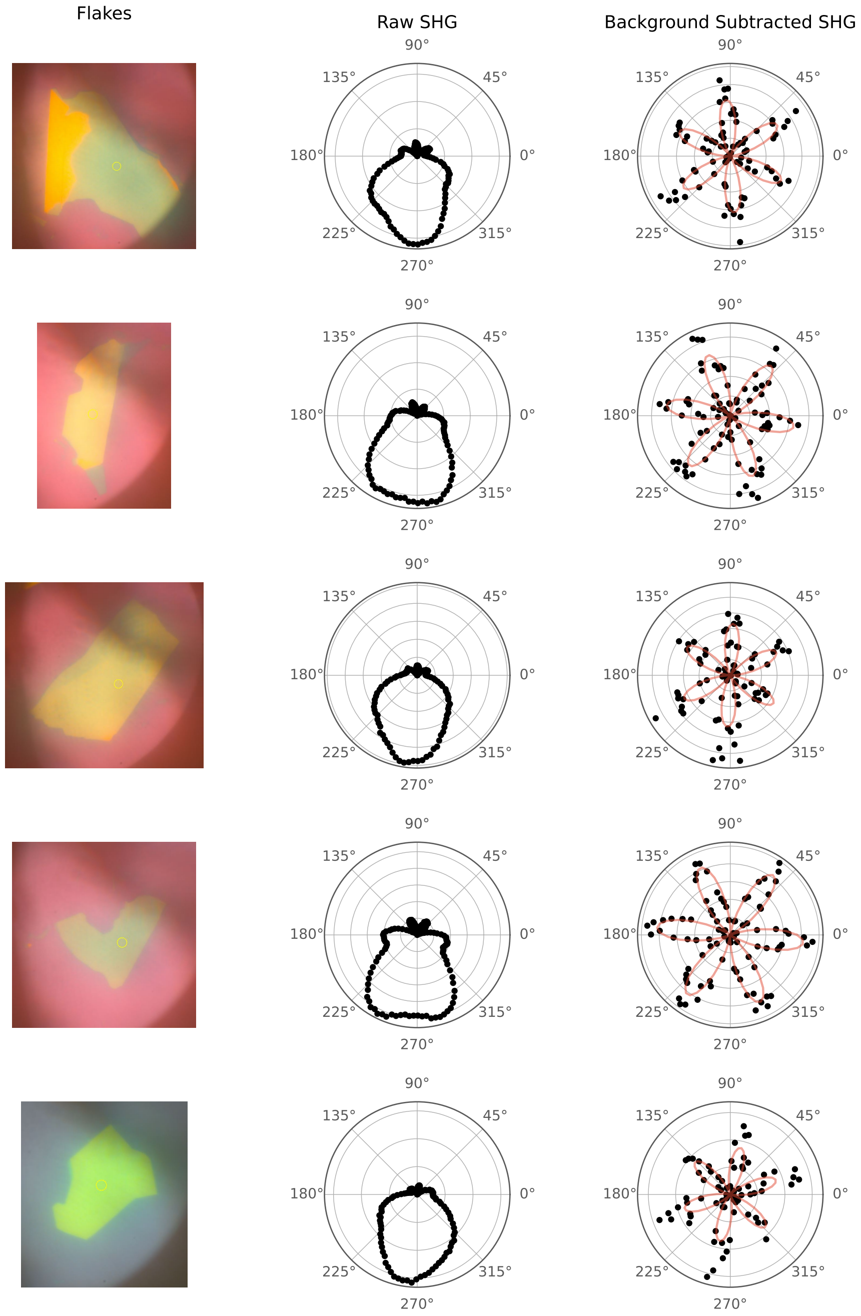

Fig. S11. Additional SHG Data SHG data taken on five hBN flakes. Left - optical image of hBN flake within the SHG measurement setup. Middle - raw SHG data for each flake, which includes SHG signal from the flake as well as an SHG signal from the measurement setup half waveplate. Right - Background subtracted SHG data with fit to $I = I_0 \cos^2(3\theta)$.

References

1. OL Blakslee, DG Proctor, EJ Seldin, GB Spence, T Weng, Elastic Constants of Compression-Annealed Pyrolytic Graphite. *J. Appl. Phys.* **41**, 3373–3382 (2003).
2. EJ Seldin, CW Nezbeda, Elastic Constants and Electron-Microscope Observations of Neutron-Irradiated Compression-Annealed Pyrolytic and Single-Crystal Graphite. *J. Appl. Phys.* **41**, 3389–3400 (2003).
3. A Bosak, M Krisch, M Mohr, J Maultzsch, C Thomsen, Elasticity of single-crystalline graphite: Inelastic x-ray scattering study. *Phys. Rev. B* **75**, 153408 (2007) Publisher: American Physical Society.
4. Y Li, et al., Probing symmetry properties of few-layer MoS2 and h-BN by optical second-harmonic generation. *Nano letters* **13**, 3329–3333 (2013) Publisher: ACS Publications.
